# Medical Journals

**Published:** 1853-10

**Authors:** 


*Medical Journals.*—*Kentucky Medical Recorder.*—Under this new title the *Transylvania Medical Journal*, formerly edited by Dr. Frasee, is to be continued, Drs. Bullit and Breckenridge, of the Kentucky School of Medicine, having assumed the editorial management. The typographical appearance of the work is improved. It is to be issued monthly as heretofore.

*The Medical Chronicle, or Montreal Monthly Journal of Medicine and Surgery.* Edited by William Wright, M. D., and D. C. MacCallum, M. D. We have inadvertently omitted to notice the appearance of this new Journal, which has reached the fourth monthly issue. It is the successor to the British American Medical Journal formerly edited by Archibald Hall, M. D.

*The Iowa Medical Journal*, is the title of a new periodical which takes the place of the North-Western Medico-Chirurgical Journal. It is issued at Keokuk, Iowa, and is conducted by the Faculty of the Iowa University.

---

Dr. Wm. O. Butler, of East Avon, intending to be absent from business for a number of months, wishes to procure an intelligent practitioner to occupy his ride during his absence. For any young man desirous of seeing practice, this affords an opportunity. Address Dr. Butler. S. B. H.

